# *Trichoderma sp.* AH pretreatment improves organic oxygen composites from supercritical alcoholysis of wheat straw

**DOI:** 10.1371/journal.pone.0315248

**Published:** 2025-03-07

**Authors:** Quanxi Zheng, Chuanyong Yan, Lei Zhang

**Affiliations:** 1 School of Chemical Engineering, Xuzhou College of Industrial Technology, Xuzhou, Jiangsu, China; 2 School of Chemical Engineering and Technology, China University of Mining & Technology, Xuzhou, Jiangsu, China; IUBAT - International University of Business Agriculture and Technology, BANGLADESH

## Abstract

Electrospray ionization fourier transform ion cyclotron resonance mass spectrometry was applied to evaluate the organic oxygen composites (OOCs) in methanol fraction derived from the supercritical alcoholysis of original and pretreated straw (WS) utilizing *Trichoderma sp.* AH. In the methanol-soluble fraction (MSF) of untreated and preconditioned WSs, *O*_n_ (n =  1–10), with double bond similar values of 1–28 and carbon atom numbers of 4–35, was the most predominant OOCs in the negative ion mode. *O*_5_ and *O*_4_ were the most prominent OOCs in the MSF of original and preconditioned WS, respectively. These results assist in comprehending the impact of *Trichoderma sp.* AH pretreatment on the specificity, and transformation of OOCs in WS alcoholysis and the application of MSF to manufacture fuel source.

## 1. Introduction

Low efficiency and specificity and low relative contents of products have limited the application of bio-oils from themolysis [1–3]. Pretreatment (PT) could decompose or disintegrate cellulose, hemicellulose, or lignin and their crystallization for efficient preparation of biomass themolysis [4–6]. Moreover, bio-pretreatment has been found to be able to enhance enzymatic scarification because of its low energy consumption, inexpensive, low reliance on chemicals, and friendly environmental factor [7–11].

So far, there are still few reports on application of fungi to promote the transformation of biological treated materials’ liquefaction, involving rice straw (RS), wheat straw (WS), soybean straw, switch grass, hardwood, and corn straw. In our previous research [12–15], supercritical alcoholysis of RS and WS could be promoted with PT utilizing *Trichoderma sp.* AH in their transformation, specially esters in methanol-soluble fraction (MSF) of WS and RS. Nevertheless, less than one hundred organic composites were identified in MSF with GC-MS. Moreover, FT-ICR-MS analysis showed that PT using *Trichoderma sp.* AH could promotes directional transformation of organic oxygen composites (OOCs) and organic nitrogen composites in supercritical alcoholysis of RS and the richness of *O*_4_ and *N*_2_*O*_4_ species in MSF could be enhanced.

In our current study, to determine effect of PT using *Trichoderma sp*. AH on OOCs produced by supercritical alcoholysis original and preconditioned WS. The FT-ICR-MS was used to analyze the MSFs of the two materials. The aim was to comprehend effect of *Trichoderma sp.* AH pretreatment on transformation of OOCs in MSF from supercritical alcoholysis of WS and the application of MSF in the manufacturing of fuel source.

## 2. Methods

### 2.1. PT of WS

WS were collected in a farm close to Yunlong District in Xuzhou, Jiangsu, China, and processed by *Trichoderma sp.* AH using techniques described in our earlier studies [12–14]. Every single one of the analytical reagents utilized as solvents had been previously purified through distillation.

### 2.2. Alcoholysis of WS

WS alcoholysis was carried out by the procedure outlined in our earlier investigations [12–14]. The approach utilized in our earlier research was utilized to calculate the outputs of gas, MSFs, and residues [12–14]. Each experiment was operated three times in order to demonstrate that the error was less than ± 5%.

### 2.3. ESI-FT-ICR-MS analysis

MSFs were determined with a Bruker apex-ultra FT-ICR MS, and detailed data was obtained by Bruker software with the method described in our earlier investigations [13,14]. Each analysis was circulated three times or more to demonstrate that the error was less than ±  5% in the relative content (RCs) of the composites found.

## 3. Results and discussion

For ease of description, MSF_5_ and MSF_0_ are utilized to denote MSF from supercritical alcoholysis of WS_5_ and WS_0_, respectively. In our earlier study [15], the highest MSF_5_ and MSF_0_ yields were 38.3% and 24.6%, respectively. The overall RCs of OOCs in MSF_0_ and MSF_5_ were 92.4% and 96.2%, respectively. As a result, negative ion ESI-FT-ICR-MS was utilized to examine the OOCs in MSF_0_ and MSF_5_, respectively.

### 3.1. Analysis of OOCs in MSF by negative ion ESI-FT–ICR-MS

**Fig 1:**
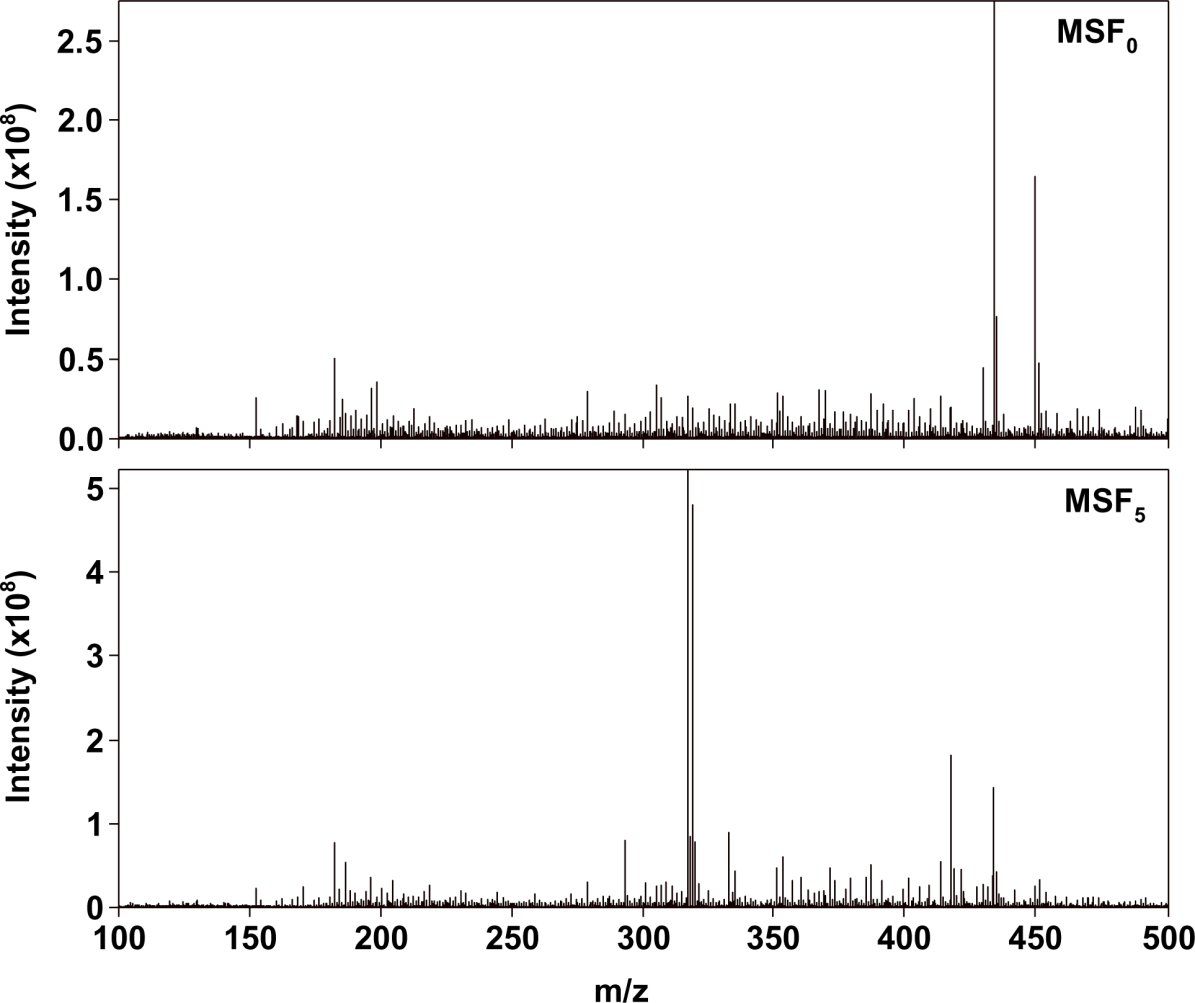
Mass spectra of MSF_0_ and MSF_5_ with analysis of negative ion ESI FT–ICR MS.

**Fig 2:**
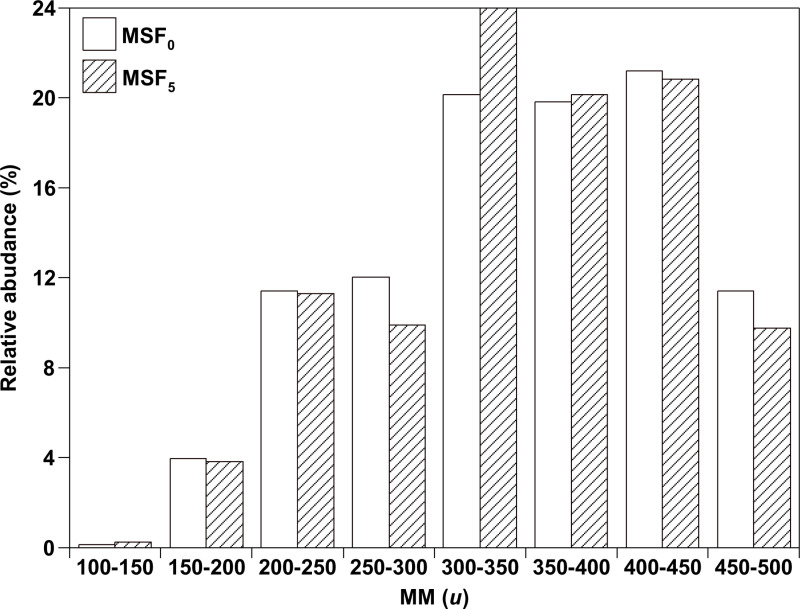
MM arrangement of compounds with analysis of negative ion ESI FT–ICR MS in MSF_0_ and MSF_5_.

MSFs had molecular masses (MMs) ranging from *m/z* 100 to 500, as illustrated in Fig 1. Observed compositions are *O*_1_–*O*_10_ and *N*_1_*O*_0_–*N*_1_*O*_9_, respectively. The masses observed for OOCs have relatively minor uncertainties in theoretical masses. According to Fig 2, the majority of MMs in MSF_5_ and MSF_0_ are distributed between 300 and 450 *u*. In comparison to MSF_0_, MSF_5_ has more relative abundances (RAs) for MMs arranged from 300 to 350 *u*.

**Fig 3:**
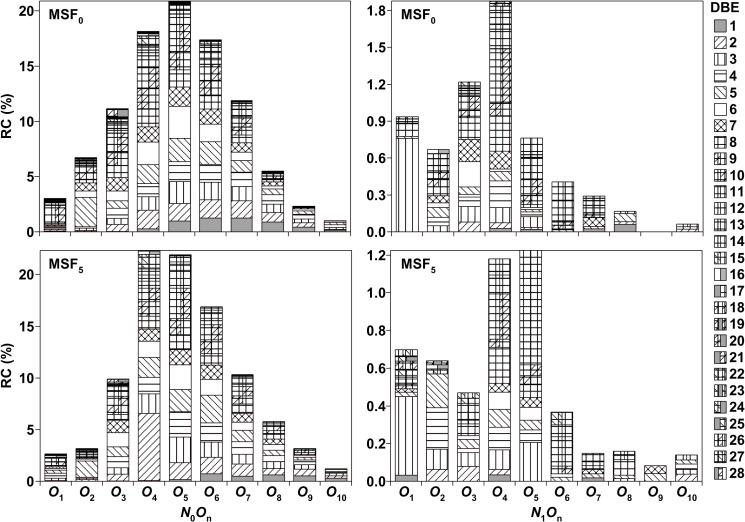
RCs of *N*_0_*O*_n_ and *N*_1_*O*_n_ species in MSF_0_and MSF_5_ with analysis of negative ion ESI FT–ICR MS.

On the basis of precise mass-to-isotope mass ratios, more than 95% of the mass spectral peaks (Fig 1) could be attributed. Ascribed elemental compositions are categorized on the basis of double bond equivalent (DBE), carbon atom number (CAN), and hetero-atom number (HAN) of our prior studies. All allocated elemental compositions are divided into the following groups: *O*_n_ (DBE =  1–26, CAN =  4–38), N_1_O_n_ (DBE =  1–22, CAN =  4–31), including *O*_1_–O_10_ and *N*_1_*O*_0_–*N*_1_*O*_9_ species. The *O*_4_ species are the most prevalent in MSF_5_, but the *O*_5_ species are the most prevalent species in MSF_0_, as revealed in Fig 3. The RCs of *O*_n_ species in MSF_5_ are 92.0%, and that of MSF_0_ are 91.3%. The distinct MMs distribution in MSF_5_ and MSF_0_ may be due to the diverse distribution of *O*_n_ in MSF_5_ and MSF_0_. These results indicate that the *O*_n_ species is the dominant compound in MSF and that its RC is significantly greater than that of the *N*_1_*O*_n_ species.

#### 3.1.1. *O*_1_ and *O*_2_ species.

**Fig 4:**
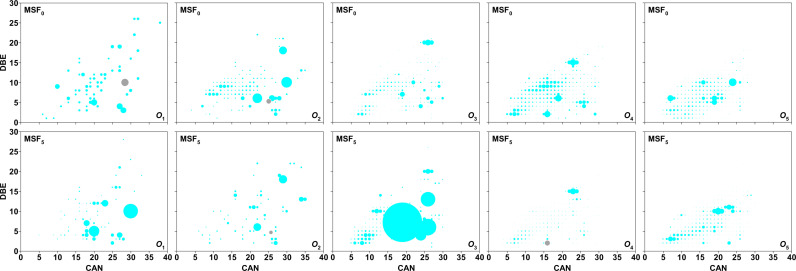
Isoabundance plots of DBE versus CAN for species *O*_1_ to *O*_5_ in MSF_0_ and MSF_5_ with analysis of negative ion ESI FT–ICR MS. The main blue–grey cycle has been replaced with grey because the relative abundance of the main blue–grey plot is so high that the size is out of frame.

**Fig 5:**
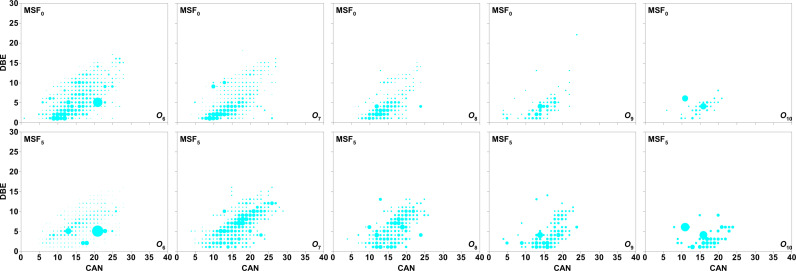
Isoabundance plots of DBE versus CAN for species *O*_6_ to *O*_10_ in MSF_0_ and MSF_5_ with analysis of negative ion ESI FT–ICR MS.

The pattern of *O*_n_ in MSF_0_ is identical to that of DBE =  1–28 and CANs =  4–38 in MSF_5_, which is equivalent to the RS that we previously analyzed, as shown in Figs 4 and 5 [14].

As shown in Fig 4, *O*_1_ species in MSF_5_ have DBEs and CANs ranging from 2 to 28 and 5 to 33, respectively, while those in MSF_0_ have DBEs ranging from 1 to 26 and CANs ranging from 6 to 38. As shown in Figs 2 and Fig 5, *O*_1_ with DBE = 10 in MSF_0_ has the highest RA, about five times that of MSF_5_, while other *O*_1_ species in MSF_0_ have relative abundances largely similar to those in MSF_5_. Most *O*_1_ species are supposed to be alkylphenols [16–18]. Alkylphenols with CAN = 15–20 are believed to be *O*_1_ species with DBE = 4 in *O*_1_, and *O*_*1*_ species with DBE = 5, 8, and 12 are supposed to be cyclohexylphenol (C_12_–C_20_), biphenyl alcohol (C_16_–C_22_) and triphenyl alcohol (C_18_–C_23_), respectively. In addition, the species in the *O*_1_ category with CANs <  18 are supposed to be biphenyl alcohols with DBE =  9, and the others are supposed to be cyclohexyl biphenyl alcohols.

The DBE and CAN for the *O*_2_ species in MSF_5_ are 2–22 and 6–35 respectively, while the DBE =  1–22 and CAN =  4–35 in MSF_0_ have similar ranges (Fig 4). As shown in Fig 2 and Fig 4, the highest RA for the *O*_2_ species in MSF_0_ is at DBE =  5 and CAN =  27, which is twice as high as in MSF_10_. Also, similar to the *O*_1_ species, the *O*_2_ species in MSF_0_ has an RA at DBE =  10 which is approximately five times higher than that of MSF_5_. A range of *O*_2_ species can be perceived at DBE =  7–11 and CAN =  8–22. The alkanoic (DBE = 1), alkenoic (DBE = 2), and alkydioic (DBE = 3) acids, which mostly correspond to stearic, hexadecanoic, and octadecadienoic acids, are thought to constitute the *O*_2_ species at DBE =  1–3.

#### 3.1.2. *O*_3_–*O*_10_.

The majority of species in the *O*_3_–*O*_10_ classes with DBE = 1–3 are supposed to be attributed to derivatives of sugar compounds [19–21], which are produced by the decomposition of cellulose or hemicellulose. As depicted in Fig 3 and Fig 4, most *O*_3_ species in MSF_5_ and MSF_0_ have CANs =  5–30 and DBE =  2–20 with the maximum RA composites in MSF_5_ having DBE = 7 and CAN = 19, about 4 times greater than in MSF_0_. Species with DBE >  3 in the *O*_3_ classes should have *p–coumarin*, coniferin, and sinapin structures, which are derived from lignin. Most *O*_4_ classes have DBE = 2–15 and CANs = 5–30, with the highest RA composites in MSF_5_ having DBE = 2 and CAN = 16, about 20 times than in MSF_0_. Based on the extents of DBE and CAN of 4–8 and 17–30 respectively, the *O*_4_ species are supposed to be Alkylphenols and esters with one or two benzene rings. The distribution and range of DBE and CAN values for the *O*_5_ species in MSF_0_ are similar to those in MSF_5_. The distribution and range of DBE and CAN in *O*_6_–*O*_10_ in MSF_5_ are also comparable to that in MSF_0_, as shown in Fig 5. With the addition of oxygen atoms, sugars become the predominant species in *O*_5_–*O*_10_.

Species with DBE = 1–3 in *O*_2_–*O*_7_ are equally numerous as that with DBE = 4–15, as seen in Figs 4 and 5. According to our prior research [14], Most of MSFs-associated acidic species are likely lignin-derived compounds, according to the comparatively high RCs in *O*_2_–*O*_9_ for DBE = 5–10 (Fig 3). Furthermore, the high RCs in *O*_3_–*O*_10_ for DBE = 1–4 suggest that the other main MSFs-associated acidic species are molecules generated from hemicellulose, and cellulose particularly sugars.

While the RCs of *O*_5_–*O*_10_ in MSF_5_ is distinct from that of MSF_0_ (Fig 3), the arrangement and range of DBE values and CAN are similar for MSF_0_ and MSF_5_, while the RCs and distribution of *O*_1_–*O*_4_ for MSF_0_ and MSF_5_ are different. As revealed in Fig 2, there are differences in the MM arrangements between MSF_0_ and MSF_5_, specially from 300 to 350 *u*. The greatest values of RC for the *O*_4_ species with DBE = 2 in MSF_5_ are much greater than those in MSF_0_, while the maximum RC values for the *O*_1_ and *O*_2_ species with DBE = 10 in MSF_5_ are lesser than those in MSF_0_ as indicated in Fig 3. These results imply that WS with BPT is inclined to be changed to *O*_4_ species through supercritical alcoholysis of WS.

PT increases the yield of OOCs and enriches *O*_4_ species in the MSF of supercritical alcoholysis feedstock and preconditioned WS. The enhancement was mainly due to structural changes in WS through PT due to incomplete decomposition with enzymes, e.g., ligninase, hemi cellulase, and cellulase. In our earlier research [15], PT utilizing *Trichoderma sp.* AH slightly altered the elemental and chemical composition of WS, and there was no significant difference in the content of the main constituents (lignin, hemicellulose, and cellulose) in original and preconditioned WS. FTIR spectra of the original and preconditioned WS showed little significant changes in bands’ spectral profiles and relative strengths, representing that lignin, hemicellulose, and cellulose were still pertain in the preconditioned WS and none of these main components disappeared considerably after preconditioning with *Trichoderma sp.* AH. SEM analysis exhibited that PT utilizing *Trichoderma sp.* AH caused prominent intrusion of gaps’ structure and formation. The structures of WS, such as lignin, hemicellulose, and cellulose, were incompletely broken down by enzymes such as ligninase, hemi cellulase, and cellulase (manganese peroxidase, laccase, and lignin peroxidase) into mono-polymers or short oligomers, so that de-polymerization and re-polymerization of hemicellulose, and cellulose with methanol can be conceded out efficiently, and easily, leading to an increased yield of OOCs and an enrichment of *O*_4_ species in MSFs.

### 3.2. Enlightened conception for efficient MSF transformation

As OOCs present in MSFs have been revealed with NIFTICRMS and they are provocative and unstable, these OOCs must been taken off or transformed when MSFs are utilized to manufacture commercial fuels. Catalytic hydro-deoxygenation and hydro-transformation are perceived to be effectient methods for superordinating Bio-oils [22–24]. A maximum of the oxygen in MSFs can be detached by catalytic hydro-deoxygenation and can be easily eliminated, while most of the nitrogen in MSFs can be easily removed by catalytic hydro-transformation. The catalytic hydro-transformation and hydro-deoxygenation of MSFs can be facilitated by biomass alcoholysis, and knowledge of the morphology of MSFs can be useful in choosing or developing effective catalysts to improve MSFs. Thus, the mixture of bio-reprocessing, alcoholysis, hydro-transformation, and catalytic hydro-deoxygenation may benefit the transformation of biomass and the application of MSFs in transport fuel production.

## 4. Summary

The range of OOCs in MSFs according to the MM distribution was from 100 to 500 u, with the majority of 300–450 u. In MSFs of raw and preprocessed WSs, are *O*_n_ (n =  1–10), DBE (n = 1–28), and CAN (n = 4–35) are the most prevalent OOCs. The most prominent OOCs in MSFs of unprocessed and preprocessed WSs are *O*_5_ and *O*_4_, respectively. In the supercritical alcoholysis of WS, PT utilizing *Trichoderma sp*. AH raised the specificity and transformation of OOCs, encouraging the concentration of *O*_4_ species in MSFs. These results help to understand how *Trichoderma sp.* AH’s PT affects the specificity, transformation, and use of OOCs in the supercritical alcoholysis of WS and the manufacture of fuel sources.

## References

[1] Prestigiacomo C, Scialdone O, Galia A. Hydrothermal liquefaction of wet biomass in batch reactors: Critical assessment of the role of operating parameters as a function of the nature of the feedstock. J Supercrit Fluids. 2022;189:105689. doi: 10.1016/j.supflu.2022.105689

[2] Wu Y, Wang H, Li H, Han X, Zhang M, Sun Y, et al. Applications of catalysts in thermochemical conversion of biomass (pyrolysis, hydrothermal liquefaction and gasification): A critical review. Renew Energy. 2022;196:462–81. doi: 10.1016/j.renene.2022.07.031

[3] Kumar R. A review on the modelling of hydrothermal liquefaction of biomass and waste feedstocks. Energy Nexus. 2022;5:100042. doi: 10.1016/j.nexus.2022.100042

[4] Selvakumar P, Adane AA, Zelalem T, Hunegnaw BM, Karthik V, Kavitha S, et al. Optimization of binary acids pretreatment of corncob biomass for enhanced recovery of cellulose to produce bioethanol. Fuel. 2022;321:124060. doi: 10.1016/j.fuel.2022.124060

[5] Mu L, Li T, Zuo S, Yin H, Dong M. Effect of leaching pretreatment on the inhibition of slagging/sintering of aquatic biomass: Ash transformation behavior based on experimental and equilibrium evaluation. Fuel. 2022;323:124391. doi: 10.1016/j.fuel.2022.124391

[6] Ouyang D, Han Y, Wang F, Zhao X. All-iron ions mediated electron transfer for biomass pretreatment coupling with direct generation of electricity from lignocellulose. Bioresour Technol. 2022;344(Pt B):126189. doi: 10.1016/j.biortech.2021.126189 34748975

[7] Dong T, Chen W, Cai C, Bai F, Zhou Z, Wang J, et al. Water-stable, strong, biodegradable lignocellulose straws replacement for plastic straws. Chem Eng J. 2023;451:138970. doi: 10.1016/j.cej.2022.138970

[8] Huang Q-S, Yan Z-F, Chen X-Q, Du Y-Y, Li J, Liu Z-Z, et al. Accelerated biodegradation of polyethylene terephthalate by Thermobifida fusca cutinase mediated by Stenotrophomonas pavanii. Sci Total Environ. 2022;808:152107. doi: 10.1016/j.scitotenv.2021.152107 34864034

[9] Lin Y, Zheng H, Dong L. Enhanced ethanol production from tree trimmings via microbial consortium pretreatment with selective degradation of lignin. Biomass Bioener. 2020;142:105787. doi: 10.1016/j.biombioe.2020.105787

[10] Baramee S, Siriatcharanon A, Ketbot P, Teeravivattanakit T, Waeonukul R, Pason P, et al. Biological pretreatment of rice straw with cellulase-free xylanolytic enzyme-producing Bacillus firmus K-1: Structural modification and biomass digestibility. Renew Energy. 2020;160:555–63. doi: 10.1016/j.renene.2020.06.061

[11] Basak B, Saha S, Chatterjee PK, Ganguly A, Woong Chang S, Jeon B-H. Pretreatment of polysaccharidic wastes with cellulolytic Aspergillus fumigatus for enhanced production of biohythane in a dual-stage process. Bioresour Technol. 2020;299:122592. doi: 10.1016/j.biortech.2019.122592 31869631

[12] Zheng Q-X, Zong Z-M, Yan H-L, Li Z-K, Kong J, Zhao M-X, et al. Supercritical methanolysis of rice straw pretreated with *Trichoderma sp*. AH. Fuel Processing Technology. 2016;154:91–5. doi: 10.1016/j.fuproc.2016.08.015

[13] Zheng Q-X, Zong Z-M, Yan H-L, Li Z-K, Wei X-Y. Improvement of organonitrogen compounds in methanol-soluble portion from supercritical methanolysis of pretreated rice straw with *Trichoderma sp*. AH. Fuel. 2017;205:100–8. doi: 10.1016/j.fuel.2017.04.110

[14] Zheng Q-X, Wei X-Y, Guo L, Xin L, Xu X, Zong Z-M, et al. Identification of organooxygen compounds in the methanol-soluble portion from the methanolysis of pretreated rice straw with *Trichoderma sp*. AH. Fuel. 2019;252:792–8. doi: 10.1016/j.fuel.2018.12.115

[15] Zheng Q-X, Wei X-Y, Xu X, Xin L, Guo L, Zong Z-M, et al. Pretreatment with *Trichoderma sp*. AH enhances conversion and specificity of wheat straw in supercritical methanolysis. Biomass Bioener. 2020;135:105149. doi: 10.1016/j.biombioe.2019.01.031

[16] Aguilar-Alarcón P, Zherebker A, Rubekina A, Shirshin E, Simonsen MA, Kolarevic J, et al. Impact of ozone treatment on dissolved organic matter in land-based recirculating aquaculture systems studied by Fourier transform ion cyclotron resonance mass spectrometry. Sci Total Environ. 2022;843:157009. doi: 10.1016/j.scitotenv.2022.157009 35772561

[17] Ware SA, Hartman BE, Waggoner DC, Vaughn DR, Bianchi TS, Hatcher PG. Molecular evidence for the export of terrigenous organic matter to the north Gulf of Mexico by solid-state 13C NMR and Fourier transform ion cyclotron resonance mass spectrometry of humic acids. Geochimica et Cosmochimica Acta. 2022;317:39–52. doi: 10.1016/j.gca.2021.10.018

[18] Dou Y, Mei M, Kettunen T, Mäkinen M, Jänis J. Chemical fingerprinting of phenolic compounds in Finnish berry wines using Fourier transform ion cyclotron resonance mass spectrometry. Food Chem. 2022;383:132303. doi: 10.1016/j.foodchem.2022.132303 35196582

[19] Calabrese V, Schmitz-Afonso I, Riah-Anglet W, Trinsoutrot-Gattin I, Pawlak B, Afonso C. Direct introduction MALDI FTICR MS based on dried droplet deposition applied to non-targeted metabolomics on Pisum Sativum root exudates. Talanta. 2023;253:123901. doi: 10.1016/j.talanta.2022.123901 36088848

[20] Ventura GT, Rossel PE, Simoneit BRT, Dittmar T. Fourier transform ion cyclotron resonance mass spectrometric analysis of NSO-compounds generated in hydrothermally altered sediments from the Escanaba Trough, northeastern Pacific Ocean. Organ Geochem. 2020;149:104085. doi: 10.1016/j.orggeochem.2020.104085

[21] Chen C, Huang Y, Wu P, Pan J, Guo P, Liu S. In vivo microcapillary sampling coupled with matrix-assisted laser desorption/ionization fourier transform ion cyclotron resonance mass spectrometry for real-time monitoring of paraquat and diquat in living vegetables. Food Chem. 2022;388:132998. doi: 10.1016/j.foodchem.2022.132998 35453011

[22] Cao D, Rao Z, Geng F, Niu H, Shi Y, Cai Y, et al. Advanced molecular-fingerprinting analysis of dissolved organic sulfur by electrospray ionization-Fourier transform ion cyclotron resonance mass spectrometry using optimal spray solvent. J Environ Sci (China). 2020;97:67–74. doi: 10.1016/j.jes.2020.05.008 32933741

[23] Islam S, Alam R, Kim S. Improved coverage of plant metabolites using powder laser desorption/ionization coupled with Fourier-transform ion cyclotron mass spectrometry. Food Chem. 2022;373(Pt B):131541. doi: 10.1016/j.foodchem.2021.131541 34810014

[24] Tian YX, Guo X, Ma J, Liu QY, Li SJ, Wu YH, et al. Characterization of biochar-derived organic matter extracted with solvents of differing polarity via ultrahigh-resolution mass spectrometry. Chemosphere. 2022;307(Pt 2):135785. doi: 10.1016/j.chemosphere.2022.135785 35870614

